# Understanding the Factors Related to Trauma-Induced Stress in Cancer Patients: A National Study of 17 Cancer Centers

**DOI:** 10.3390/ijerph18147600

**Published:** 2021-07-16

**Authors:** Matthew R. Moore, Cindy Davis, Tamara Cadet, Tina Harralson, Laura Dietzen

**Affiliations:** 1 School of Social Work, College of Behavioral and Community Sciences, University of South Florida, Tampa, FL 33620, USA; moorem2@usf.edu; 2 School of Law and Society, University of the Sunshine Coast, Queensland 4558, Australia; 3 School of Social Policy and Practice, University of Pennsylvania, Philadelphia, PA 16802, USA; tamara.cadet@simmons.edu; 4 Tridiuum, Inc., Philadelphia, PA 19103, USA; T.Harralson@Tridiuum.com (T.H.); L.Dietzen@Tridiuum.com (L.D.)

**Keywords:** cancer care, distress, oncology, posttraumatic stress, psychosocial, social support, survivorship

## Abstract

Objective: Posttraumatic stress symptoms (PTSS), defined as continued trauma, has been found to negatively impact mental and physical health. Many cancer centers routinely assess level of psychological distress but assessment of symptoms related to PTSS is less routine. Understanding the mechanisms by which psychological distress results in, or influences, PTSS will aid in developing protocols to more effectively identify PTSS in cancer patients. Methods: Survey data were analyzed from intake data at 17 cancer centers across the U.S. Patients reported distress ratings on the National Comprehensive Cancer Network (NCCN) Distress Thermometer (DT), responded to questions related to intrusive cognitive symptoms of PTSS and provided information about current symptoms and social support systems. Hypotheses were tested using a conditional process model, and paths were provided for direct and indirect effects, including moderation and mediated moderation. Results: Findings indicated that, while distress scores were influential in the total model, the direct effect of distress on intrusive cognitive symptoms of PTSS was negated by the model’s indirect effects. The effects of social support and older age were independent protective factors, and there was a moderation effect that varied across groups. Lastly, physical cancer symptoms as a mediating variable further explained the relationship between psychological distress and intrusive cognitive symptoms of PTSS. Conclusions: Study results provide evidence for a potential mechanism by which distress relates to intrusive cognitive symptoms of PTSS. Furthermore, findings suggest that older age and social support may be protective factors for certain groups and risk factors for others. This study provides formative data for potential next steps that could lead to improvements in routine psychosocial screenings in cancer treatment settings.

## 1. Introduction

Cancer diagnosis and treatment is a stressful process, with effects that have been compared to those seen in individuals who have experienced active combat exposure [1,2]. In response to these stressors, cancer patients and caregivers frequently contend with severe psychological distress, as well as posttraumatic stress disorder (PTSD) or posttraumatic stress symptoms (PTSS) [3]. There are significant differences between PTSD and PTSS. PTSS refers to the symptoms associated with a traumatic experience; whereas, PTSD is a psychiatric disorder that involves more severe and chronic symptoms related to a traumatic experience. PTSS is a continuous response due to traumatic events and occurs due to chronic stress. Trauma can be considered a severe shock characterized by disturbances in one’s defense mechanisms and the loss of all functions of the body [4,5]. PTSS is caused by intrusive thoughts contributing to the inability of an individual to address an unpleasant experience as a result of a cancer diagnosis and/or treatment [5].

In 2021, estimates indicate 1.9 million new cancer cases with approximately 17 million cancer survivors [6]. Psychological distress (or distress as will be referred to in this paper) has been recognized as the “sixth vital sign”, and as such, is a primary psychological concern in cancer care [7,8]. There is a continuum of distress that ranges from normal distress (e.g., sadness, vulnerability, fear) to severe distress (e.g., depression, anxiety, panic, social isolation). Severe distress is often found in cancer patient populations [7]. In response to this concern, the American College of Surgeons’ Commission on Cancer required that all accredited cancer centers implement the monitoring of distress in routine patient care by 2015. As a result, many treatment centers have started using tools such as the National Comprehensive Cancer Network (NCCN) Distress Thermometer (DT) in daily practice. The DT is frequently used due to its utility, ease of use, and brevity [7,9]. In a study of 4664 cancer patients in 55 cancer centers in North America, 46% of patients were found to experience significant distress as measured by the DT [9].

Not surprisingly—like distress—cancer patients have been found to experience symptoms of PTSS at rates significantly higher than those found within the general public [10,11,12]. It has been found that PTSD ranges between 5% and 19% for cancer patients, with an estimated 5–13% additional cancer patients experiencing PTSS [12]. Although effective methods for identifying traumatic stress exist, the associated costs and patient participation often make additional or intensive assessment unrealistic, resulting in cancer centers screening simply for overall distress using the DT [9]. Thus, understanding the relationship between PTSS and a commonly-assessed domain such as distress could help improve future detection of PTSS in cancer patients.

Untreated chronic distress and PTSS have been linked to increased risk for other mental health problems and the onset of health concerns such as cardiovascular disease. There are various factors related to PTSS that serve as risk and protective factors, such as, isolation, social support, and pre-existing schema that can contribute to negative health outcomes [13,14]. Furthermore, distress and PTSS may not abate without appropriate psychological treatment [1,13]. Research has documented efficacious interventions for cancer patients experiencing severe distress or traumatic stress, and the importance of beginning these treatments as early as possible [15,16]. Understanding the role of distress and PTSS, along with their relationship to the stress resulting from cancer is critical to improving patient care and outcomes [13,14]. Thus, the need to understand these relationships suggests that the identification of symptoms related to both distress and PTSS should be considered an essential piece of routine cancer care.

There is limited evidence to understand the factors related to distress and PTSS in cancer patients. Findings from a systematic review and meta-analysis indicate a strong positive association between distress and PTSS (*r* = 0.62) from 8 studies with all studies reporting positive associations that were moderate to strong (range *r* = 0.41 to 0.76) [2]. In addition, this meta-analysis found that PTSS were associated with depression (*r* = 0.56), anxiety (*r* = 0.65), social support (*r* = −0.33), and physical quality of life (*r* = −0.44) [2]. More recent studies also suggest a positive relationship between PTSS, physical symptoms, and emotional distress in cancer survivors [17,18]. Physical cancer symptoms have also been linked to distress, depression, and long-term anxiety, which suggests a possible relationship between physical symptoms, distress, and anxiety-related concerns such as PTSS [17,18,19]. Subsequent and recent studies focus on children and their parents and note that the lack of evidence that explains the pathways between distress and PTSS [20,21]. Previous evidence indicates that age and social support are moderators of distress and PTSS [2,18]. Specifically, age has been found to moderate the relationship between cancer-related distress and PTSS [3]. Social support has been frequently identified as a potential protective factor for significant distress, which is, in turn, related to traumatic stress [2,3,22,23]. Although social support has been linked to both distress and PTSS, research has also indicated that social support may not be a direct predictor of PTSS [24].

Given the lack of evidence to understand the factors of distress and PTSS that affect cancer populations and the need to determine how to best support cancer patients who are experiencing distress but may also be experiencing other symptoms, this study examines the multiple pathways that can help practitioners determine appropriate steps to ultimately decrease PTSS symptoms. Currently, the literature independently suggests that social support, age, distress, and physical symptoms are related to PTSS. However, since PTSS is about the intrusive re-experiencing of the traumatic event or intrusive cognitive thoughts, it is critical to first examine the possible pathways prior to enhancing, suggesting, or developing interventions that focus on PTSS, not PTSD.

**Figure 1:**
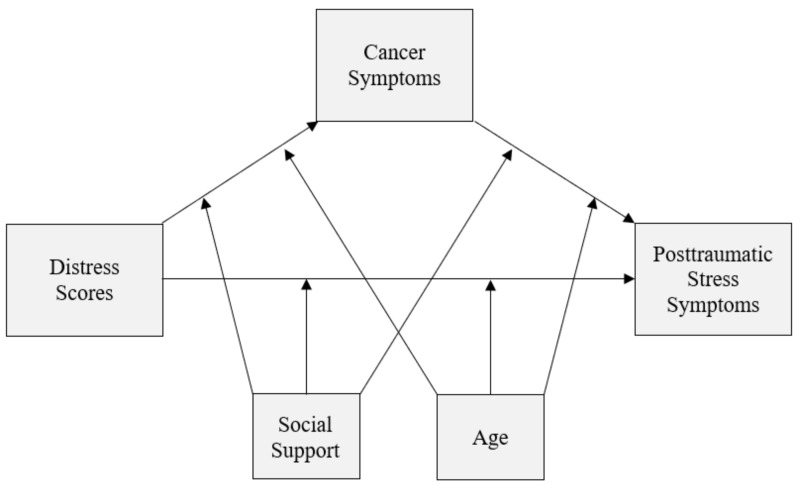
Conceptual diagram of conditional process model.

Based on factors identified in the literature [2,3,17,18,19,20,21,22,23,24], the purpose of this study was to examine a hypothesized path model (see Figure 1) that suggests a relationship between distress and intrusive cognitive symptoms of PTSS, by way of physical cancer symptoms. As age and social support are the most frequently cited moderators of distress and intrusive cognitive symptoms of PTSS, they were also included in the model. The following hypotheses were evaluated:

**Hypothesis 1** **(H1).** *Increased levels of psychological distress are associated with higher levels of intrusive cognitive symptoms of PTSS.*

**Hypothesis 2a** **(H2a).** *Older age will reduce the effect of distress on intrusive cognitive symptoms of PTSS.*

**Hypothesis 2b** **(H2b).** *Higher levels of social support will reduce the effect of distress on intrusive cognitive symptoms of PTSS.*

**Hypothesis 3** **(H3).** *The relationship between distress and intrusive cognitive symptoms of PTSS will be mediated by physical symptoms.*

**Hypothesis 4a** **(H4a).** *The effect of distress on physical symptoms will be moderated by age.*

**Hypothesis 4b** **(H4b).** *The effect of distress on physical symptoms will be moderated by social support.*

**Hypothesis 5a** **(H5a).** *The mediation effect will be moderated by age.*

**Hypothesis 5b** **(H5b).** *The mediation effect will be moderated by social support.*

## 2. Methods

### 2.1. Data and Sample

This study used secondary data collected as part of routine intake procedures at 17 cancer centers, located in nine states across the continental U.S. [24]. This sample included patients from geographically diverse areas and included clinics located in rural, suburban, and urban areas. During intake, patients were asked to complete an electronic psychosocial assessment, and data were collected by the health informatics company. This assessment included questions regarding current cancer symptoms, available social support, mental health indicators such as levels of psychological distress and intrusive cognitive symptoms of PTSS. The assessment was completed on a tablet device, and questions were available in both English and Spanish. This paper is a secondary analysis of deidentified data [24]. The data was secondary data and received without identifying information so there was not a process to obtain consent. De-identified data were provided to the investigators and approval for secondary data analysis was obtained from the University of Tennessee institutional review board (UTK IRB-17-03783XM).

### 2.2. Measures

#### 2.2.1. Distress Thermometer (DT)

The DT was utilized in gathering self-report information about each patient’s level of psychological distress. Participants were asked to rate their level of distress in the past week, from 0 to 10, with 10 indicating severe distress. This variable was identified as the primary predictor for statistical modeling purposes. Akizuki et al. reported sensitivity and specificity values for the DT at 0.84 and 0.64, respectively [25]. Similar values have been found in other studies as well [26].

#### 2.2.2. Posttraumatic Stress Symptoms (PTSS)

Three questions were asked to identify intrusive cognitive symptoms of PTSS. Previous studies support the importance of examining intrusive cognitive thoughts in relation to psychosocial functioning and PTSS in cancer patients [27,28,29]. These questions were: “In the past two weeks, how often have you felt like you are reliving your diagnosis of cancer?”, “In the past two weeks, how often have you had “nightmares” or “disturbing dreams” about your cancer?” and “In the past two weeks, how often have you been bothered by intrusive thoughts or images about your cancer?” The items were scored on a Likert-type scale, from “never or rarely” to “all or most of the time.” Responses were totaled to create a sum of PTSS symptoms with scores ranging from zero to twelve with higher scores indicating more intrusive cognitive symptoms of PTSS, which was used as the primary outcome variable in this study. Content validity of these items in relation to the subscale of intrusive cognitive symptoms Criterion B on the DSM5 was established by comparison to subscales from a standardized scale [30,31].

#### 2.2.3. Physical Symptoms

Cancer-related physical symptoms were also included in the assessment. Patients were asked to give a series of binary (yes/no) responses to indicate if they had experienced problems with pain, tiredness or fatigue, nausea or vomiting, sexual dysfunction or lack of interest in sex, somatic disturbances, or bowel difficulties. These responses were combined into a cancer symptom score, where lower values indicate more severe symptomology.

#### 2.2.4. Age and Sex

Age was measured in years and sex was measured dichotomously—male and female.

#### 2.2.5. Social Support

Social support levels were assessed with ordinal-scaled items, with possible responses ranging from “strongly disagree” to “strongly agree”, with five total response categories. Respondents were asked if they had friends or family to: help with life responsibilities, help with transportation to treatment, help with financial concerns, to ask for advice, to provide emotional support, to provide comfort and understanding, or to help with the overall difficulty of cancer treatment. Item responses were summed to create a total score representing social support, with higher values indicating higher levels of positive social support. Content validity of these items was established by comparison to a standardized scale [32].

### 2.3. Data Analysis

Data were analyzed using IBM Statistical Package for the Social Sciences (SPSS), version 24.0. Univariate descriptive statistics and bivariate correlations were assessed prior to model construction, as were the regression model assumptions. A power analysis was conducted by utilizing Fritz and Mackinnon’s estimates for required sample sizes when detecting indirect effects. It was determined that a sample size of 558 would be sufficient for detecting small effects, given a power of 0.80 [33].

Upon completion of data screening, a conditional process analysis was conducted using Hayes’ Model 76 in the PROCESS SPSS macro to test the study hypotheses. This conditional process model was selected, as it allows for the examination of complex relationships through the simultaneous testing of both direct and indirect effects [34]. In this study, we utilized the model to examine the direct effect of distress on intrusive cognitive symptoms of PTSS when moderated by age and social support, as well as the indirect effects of this relationship by way of the potential mediating influences of physical health symptoms.

Scores from all multi-item scales were assessed to determine if there was sufficient internal consistency using Cronbach’s Alpha. Reliability analyses were conducted and there were no problematic items identified. Coefficient estimates indicated sufficient reliability for the intrusive cognitive symptoms of PTSS, physical symptoms, and social support scales, with Cronbach’s Alpha values of 0.70, 0.89, and 0.69, respectively.

## 3. Results

*Sample:* Responses for 1140 patients were recorded. Participants with missing values on any of the predictor or outcome variables were removed listwise, as a function of the conditional process analysis. The deleted cases included any participant with a missing value in any of the study variables. These cases were analyzed to determine if there were any important differences between their responses on other study variables and the remaining sample. No differences were identified and the omission of these cases proved inconsequential to the attainment of the desired statistical power, so imputation was not used. This left a total of 1119 cases in the final sample. To better describe the analyzed data, descriptive statistics were analyzed using the reduced sample. Demographic information captured in this study was limited to age and sex. Participants ranged in age from 18 to 93 (*M* = 60.58, *SD* = 12.84). The sample consisted of 31.8% male and 68.2% female patients. Demographic characteristics are displayed by cancer center in Table 1. More than twelve types of cancer were represented in these data, with breast cancer (33.2%) and lung cancer (11.3%) as the largest groups. However, this variable was excluded from analysis, because 35% of participants did not specify a cancer type. Seventy-eight percent of participants responded with a “4” or higher on the DT, indicating significant distress (*M* = 5.07, *SD* = 2.70). Though the majority of patients met this clinical cut-off, DT scores have been found to vary greatly, with rates regularly reported from 30% to 70% [11,35,36,37]. The percentage of participants indicating some degree of PTSS was 18.2%, which is within the ranges found in other studies [14].

Bivariate analysis: Bivariate correlations indicated a moderate correlation between distress scores and intrusive cognitive symptoms of PTSS (*r* = 0.377, *p <* 0.001). There was also a significant negative correlation between distress scores and the physical cancer symptoms scores (*r* = −0.669, *p <* 0.001), as well as distress and social support (*r* = −0.350, *p <* 0.001), indicating that higher levels of distress and lower levels of social support were related to more physical symptoms. A small, but significant negative correlation was found between distress and age (*r* = −0.091, *p* = 0.002) indicating a relationship between higher distress and younger participants. Intrusive cognitive symptoms of PTSS were also correlated negatively with symptom scores (*r* = −0.523, *p <* 0.001), social support (*r* = −0.077, *p <* 0.001), and age (*r* = −0.125, *p <* 0.001), indicating that more cognitive symptoms of distress were related to more physical symptoms, less social support and younger age. Sex was not significantly correlated with any of the study variables, except age (*r* = −0.195, *p <* 0.001). Correlations and mean values for all study variables are shown in Table 2.

Conditional process analysis: In Step 1 of the conditional process model, distress was regressed on the proposed mediator, cancer symptoms. Social support and age were added as moderators. To control for the effect of sex, sex was added to the model as a covariate. This intermediate model explained nearly 50% of the variance in symptom scores (*R*^2^ = 0.491, *F*(6,1112) = 179.07, *p <* 0.001). Distress was directly related to symptom scores (*t*(6,1112) = −3.48, *p* < 0.001), as were social support (*t*(6,1112) = 8.40, *p* < 0.001), and the interaction effect between distress and social support (*t*(6,1112) = −7.73, *p* < 0.001).

**Figure 2:**
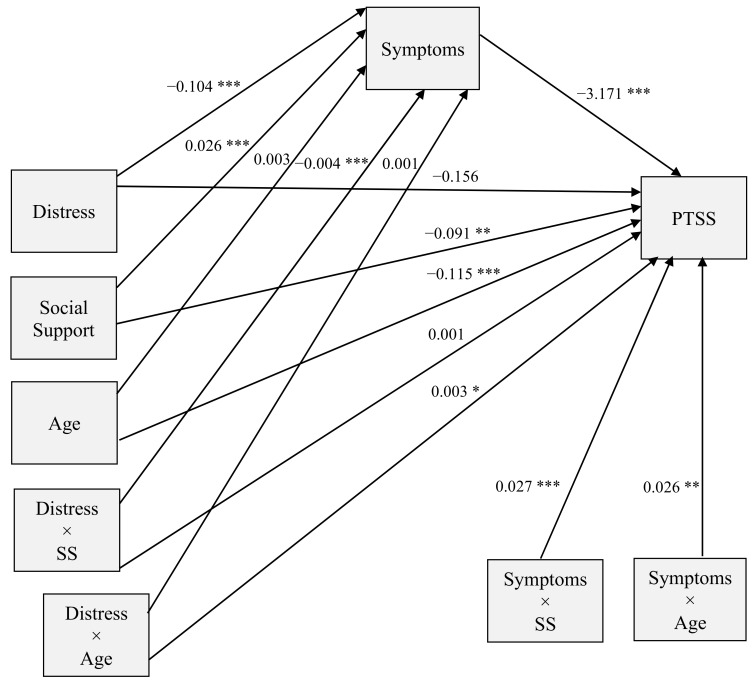
Path diagram and coefficient values for conditional process model. Note. * *p* < 0.05 ** p < 0.01 *** *p* < 0.001.

In Step 2, the model accounted for the direct effect of distress on intrusive cognitive symptoms of PTSS, as well as the mediation, moderation, and mediated moderation effects. The final model accounted for 31% of the total variation in intrusive cognitive symptoms of PTSS (*R*^2^ = 0.310, *F*(9,1109) = 55.42, *p <* 0.001). Cancer symptom scores predicted intrusive cognitive symptoms of PTSS (*t*(9,1109) = −8.48, *p <* 0.001), as did the interaction between symptoms and social support (*t*(9,1109) = 4.07, *p <* 0.001) and age (*t*(9,1109) = 4.25, *p <* 0.001). Similarly, social support alone (*t*(9,1109) = −3.09, *p* = 0.002), age alone (*t*(9,1109) = −4.12, *p <* 0.001), and the distress/age interaction (*t*(9,1109) = 2.13, *p* = 0.034) were statistically significant predictors of intrusive cognitive symptoms of PTSS. Specifically, increases in age or social support were related to decreased levels of intrusive cognitive symptoms of PTSS. Mediation was observed, as distress did not have a direct effect on intrusive cognitive symptoms of PTSS (*t*(9,1109) = −1.51, *p* = 0.131) when controlling for the other variables. As was the case with the direct effect of distress on intrusive cognitive symptoms of PTSS, the distress and social support interaction did not have a significant effect on intrusive cognitive symptoms of PTSS (*t*(9,1109) = 0.722, *p* = 0.470). Figure 2 represents all paths in the conditional process model, with coefficient values listed for each relationship.

Significant moderation was demonstrated in the conditional direct effects of distress on intrusive cognitive symptoms of PTSS. Specifically, this relationship was moderated for patients with one standard deviation above the mean age, at all levels of social support. The strongest effects were shown in patients at one standard deviation above the mean age, at both mean (*p* < 0.001, 95% CI [0.056, 0.171]) and one standard deviation above mean levels of social support (*p* < 0.001, 95% CI [0.061, 0.192]). Moderation also occurred at one standard deviation below mean social support levels and one standard deviation above the mean age (*p* = 0.012, 95% CI [0.022, 0.173]), and in those at mean age with mean levels (*p* = 0.001, 95% CI [0.028, 0.112]) and above mean levels of social support (*p* = 0.003, 95% CI [0.029, 0.143]). Examination of this direct effect moderation indicates that increased levels of social support and at-or-above mean age are related to an increase in the positive relationship between distress levels and intrusive cognitive symptoms of PTSS. This means that, although older age and increased social support were associated with decreased levels of intrusive cognitive symptoms of PTSS, the buffering effect of social support may not apply to older adults.

Moderated mediation occurred at all levels of the moderating variables, as shown in Table 3. Effects were evaluated by bootstrap estimation, with confidence intervals generated based upon 5000 samples. Statistically significant relationships were indicated by confidence intervals not containing zero. Results indicated that as levels of social support rise, the difference between age groups increases. The largest effects occurred when social support levels were one standard deviation below mean age and at (*b* = 0.228, *SE* = 0.024, 95% CI [0.181, 0.276]), or one standard deviation above (*b* = 0.235, *SE* = 0.035, 95% CI [0.166, 0.305]) mean levels of social support.

**Figure 3:**
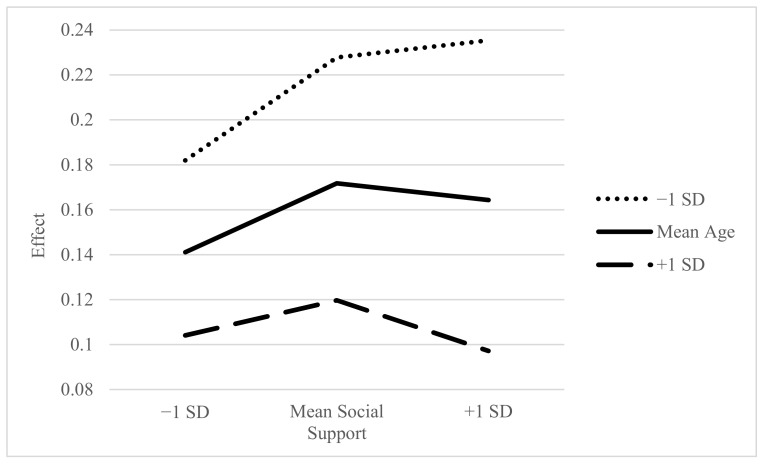
Conditional Indirect Effects of Distress on PTSS as Moderated by Age and Social Support (Mediated by Symptom Score).

Unlike the results seen in the direct effect moderation, this moderated mediation indicates that—when considering the physical symptoms associated with cancer—changes in age and social support affect the severity of PTSS across all groups. Specifically, the strongest indirect effects were seen in younger individuals who reported higher than average levels of social support. These increased effects would serve to reduce the mediating influence of physical symptoms on PTSS and indicate that social support is less of a protective factor in younger individuals. This is further illustrated in Figure 3.

## 4. Discussion

The results supported seven of the eight hypothesized relationships. Findings from the conditional process model indicated that increased levels of distress are predictive of intrusive cognitive symptoms of PTSS, but that this relationship may be a function of physical symptoms. Both older age and increased levels of social support were shown to decrease intrusive cognitive symptoms of PTSS. Nonetheless, the combined moderation effect of these variables on the relationship between distress and intrusive cognitive symptoms of PTSS achieved an increase in intrusive cognitive symptoms of PTSS for older adults with higher levels of social support. Social support also functioned as a moderating influence on the distress to symptom score path, but the hypothesized moderation of age was not supported. Additionally, the observed mediating effect of physical symptoms was conditional upon levels of age and social support. This moderation was reflective of higher intrusive cognitive symptoms of PTSS in younger patients with moderate to high levels of social support when considering the physical symptom mediation.

Interestingly, physical symptoms not only mediated the relationship between distress and intrusive cognitive symptoms of PTSS, but the direct relationship between distress and intrusive cognitive symptoms of PTSS was significant as well. Specifically, increased distress was related to more physical symptoms. This was consistent with previous studies, which have reported similar findings around the notion that distress is often accompanied by an increase in physical symptoms [2,37,38]. Previous studies have also found a significant relationship between PTSS and physical symptom and depressive symptomology [17,39].

In sum, key findings are as follows:

- Both older age and increased levels of social support were shown to decrease intrusive cognitive symptoms of PTSS. This suggests that those individuals who were older and those with higher social support had less intrusive cognitive symptoms.
- The combined moderation effect of age and social support on the relationship between distress and intrusive cognitive symptoms of PTSS achieved an increase in intrusive cognitive symptoms of PTSS for older adults with higher levels of social support. This suggests the combination of older age and higher social support increased the intrusive cognitive symptoms.
- Social support also functioned as a moderating influence on the distress to symptom score path. Evidence indicated that social support may have served to attenuate the relationship between distress and PTSS symptoms.
- The hypothesized moderation of age was not supported. While previous literature has indicated that age may influence PTSS, it was not shown to have an independent effect in this study. This moderation was reflective of higher intrusive cognitive symptoms of PTSS in younger patients with moderate to high levels of social support. When combined with physical symptom mediation, younger age and higher levels of social support had an increasing effect on this mediating relationship.
- There was a direct relationship between distress and intrusive cognitive symptoms of PTSS, but the study also found that physical symptoms mediated the relationship. This suggests that while distress and PTSS may be related, the mechanism by which this relationship functions may be more complicated than it appears. Specifically, the pathway by which distress is related to intrusive cognitive symptoms of PTSS can occur by way of the physical symptoms associated with cancer treatment. The observed mediating effect of physical symptoms was conditional upon levels of age and social support. This suggests variability in the relationship between physical symptoms and PTSS, based upon an individual’s age and level of social support.

This study provides important evidence for the potential relationship between distress and intrusive cognitive symptoms of PTSS, as well as a better understanding of how age and social support may help insulate certain groups from the deleterious effects of high levels of psychological distress, and how the physical effects of cancer can alter this relationship. In addition, much of the literature on distress and intrusive cognitive symptoms of PTSS in cancer patients is limited to homogenous groups. Often, studies are conducted with samples limited to one type of cancer or sex, which limits generalizability. This study is derived from a sample of patients with numerous types of cancer, and although not evenly split by sex, is more than 30% male.

### 4.1. Study Limitations

This study was cross-sectional and utilized a secondary data source of self-reported data. As such, there were several variables that were not available for analysis. In particular, this study was limited in its ability to assess some key demographic variables: ethnicity, education, or socioeconomic status. As income has been previously reported as a significant predictor of distress, its omission from the data must be considered a limitation [40]. Additionally, though these data represent a variety of cancer diagnoses, the large amount of missing data in the cancer type variable limited its utility in this study.

It has been suggested that specificity in identifying the type of social support is important when determining its relationship with mental health outcomes in healthcare settings. The data in this study were limited to positive social support. However, studies have shown that negative social support may also be significantly related to depression and distress [41,42]. Moreover, social support may need to be more clearly defined and measured with a standardized scale in future studies when establishing a link to PTSS or distress [23]. Similarly, the measure for PTSS was not intended to address all of the five DSM-5 symptom clusters—rather it was intended to only assess intrusive cognitive symptoms (Criterion B), which is a limitation of the study. The use of non-standardized measures is a noted limitation of the current study, and the measures used for the cognitive symptoms of PTSS and social support may have only captured a narrow view of these concepts as opposed to more comprehensive measures of these domains. Future studies should consider a comprehensive and standardized measure of PTSS and social support.

Additionally, the DT was used as an indicator of distress in this study. Although the DT has been praised for its value within clinical settings, it was developed as a screening tool. Its use as a diagnostic instrument may be suspect and should be considered when evaluating the findings of the current study [43,44]. Restriction of range must also be considered with these data. Indicators of physical symptoms, intrusive cognitive symptoms of PTSS, and social support are measured on an ordinal scale. In addition, DT scores are recorded as zero to ten responses, and the mean was nearly six. Restriction of range may result in weakened correlations and biased findings.

### 4.2. Research Implications

Future studies should further examine the relationship between distress and PTSS. This could include examining the utility of the DT in identifying PTSD or PTSS. While this study was limited to three indicators of intrusive cognitive symptoms of PTSS and although a link between DT scores and intrusive cognitive symptoms of PTSS was identified, additional items of measures of PTSS would allow for more in-depth investigation in the future. This could also include standardized PTSD measures, though as mentioned earlier, partial PTSS are more commonly reported than clinical PTSD. In addition, researchers should examine the effect of social support on the relationship between distress and PTSS, with added specificity in the various domains of social support.

The mean distress rating given in this sample was above that traditionally considered as the cut score for clinically significant distress. While the body of research generally supports the use of the DT as a screening instrument, the elevated distress levels reported in this sample indicate the potential need for additional research to examine the cut score that would provide optimal sensitivity and specificity. Additionally, if the DT is to be used as a potential screening instrument for other mental health concerns such as PTSS, a cut score should be established to represent those domains.

Perhaps most importantly, this study presents the possibility that, although distress and PTSS appear to be linearly related, their relationship may be a function of another variable. Specifically, this study found that, when considering physical cancer symptoms, there was no direct relationship between distress and intrusive cognitive symptoms of PTSS. Findings from the current study suggest variability in the relationship between physical symptoms and PTSS, based upon an individual’s age and level of social support. Further research should examine whether this variability is related to the possible increased burden of physical limitations on older adults and the potential greater need for social support, as compared to their younger counterparts.

### 4.3. Clinical Implications

Research indicates that the most effective way to identify traumatic stress in cancer patients is to conduct multidimensional assessments at different times and in different ways [2,43]. Unfortunately, conducting assessments in that way can be very costly and time-consuming, and simply not practical in many clinics. Not surprisingly, the clinics that are unable to outlay the resources needed are often those that treat the underserved. In light of this reality, it is important to identify those most at risk and to improve screening procedures so that they are more efficient and maximize available resources. This includes utilizing current measurement instruments in new and innovative ways. The DT has been shown in previous research to be an effective tool for not only identifying clinical distress but also the symptoms of other depressive and anxiety-related disorders [25,45]. Given the findings of the current study, as well as the fact that PTSD is also an anxiety disorder and its symptoms have been previously connected to distress levels, it stands to reason that there could be some potential overlap in the screening utility of this instrument. Clinicians should bear in mind that elevated scores on the DT may indicate a greater possibility for PTSS, so patients reporting extreme distress scores should be assessed for PTSS [8]. This would provide an indicator for clinicians in centers without the resources to screen every patient for PTSS.

Additionally, clinicians should consider the screening utility of tools such as the DT. While the DT may provide adequate sensitivity, the specificity may be less desirable. A large portion of the patients surveyed in this study reported increased levels of distress. Improperly attributing clinical distress to patients may result in the misallocation of resources and unneeded treatment for the patient.

Clinicians should also understand that, although older patients may be less likely to experience distress or PTSS separately, the mechanism by which one transitions from distress to PTSS may be altered by age. Similarly, social support may provide a buffer for the stress of cancer, and it may complicate the experience for certain groups. If this is the case, then clinicians should consider that programs designed to enhance social support networks may be helpful for some patients and potentially harmful for others. Similarly, younger patients largely experienced increased levels of distress. Clinicians should consider expanding assessment protocols for at-risk groups, such as younger cancer patients. This could include ensuring that these patients complete the DT’s 39-item problem checklist and quickly identifying any reported problem areas.

Cancer patients should be made aware of the potential link between distress and PTSS and encouraged to share their symptoms with their providers as early as possible. Studies have also found a relationship between PTSS and depressive symptomology [17,39]. Although treatments can be effective for distress and PTSS, early detection and treatment are important. If cancer patients are educated on the long-term consequences of chronic distress and the increased odds of developing PTSS, then they may be more likely to avail themselves of needed services. These findings provide an important opportunity to improve cooperation among multidisciplinary cancer teams. Studies have consistently shown that prolonged, untreated distress and PTSS can contribute to stress-related negative health outcomes [1]. Moreover, untreated distress and PTSS can worsen treatment outcomes by altering patient health behaviors such as treatment adherence [32,46,47]. Social workers, psychologists, nurses, and physicians all have a role to play in identifying risk factors and screening for mental health issues, with effective communication between the team being essential to make patients less likely to experience distress or PTSS. However, it is important to address institutional barriers that might reduce the likelihood of implementing screening procedures and using standardized instruments in the screening process [48,49,50].

## Figures and Tables

**Table 1:** Sample Characteristics by Center.

			Sex		Age	
Center (*n* = 17)	Setting (Non-Rural = 76%) (Rural = 24%)	*n* (1119)	Male (31.4%)	Female (68.6%)	M (60.58)	SD (12.84)
1	Non-rural	210	31.90	68.10	61.14	11.62
2	Rural	58	36.20	63.80	63.57	13.94
3	Non-rural	10	40.00	60.00	55.00	13.00
4	Non-rural	17	0.00	100.00	53.18	12.84
5	Non-rural	15	40.00	60.00	54.27	13.44
6	Non-rural	5	80.00	20.00	67.60	10.78
7	Non-rural	9	11.10	88.90	47.00	20.12
8	Non-rural	39	30.80	69.20	62.13	14.36
9	Non-rural	24	29.20	70.80	50.58	12.00
10	Non-rural	21	0.00	100.00	56.71	15.06
11	Rural	236	33.10	66.90	59.14	12.47
12	Rural	107	23.40	76.60	61.29	12.34
13	Non-rural	90	47.80	52.20	58.79	11.01
14	Non-rural	22	22.70	77.30	55.95	14.10
15	Non-rural	30	40.00	60.00	62.23	12.52
16	Rural	35	22.90	77.10	62.71	11.55
17	Non-rural	191	30.40	69.60	64.4	12.74

**Table 2:** Correlations and Descriptive Statistics for Key Study Variables.

Bivariate Correlations and Descriptive Statistics (*n* = 1119)								
	1	2	3	4	5	6	M	SD
1. Distress	-						5.07	2.70
2. PTSS	0.377 ***	-					4.32	1.63
3. Symptoms	−0.669 ***	−0.523 ***	-				3.71	0.70
4. Social Support	−0.350 ***	−0.077 **	0.299 ***	-			17.54	13.39
5. Age	−0.091 **	−0.125 ***	0.153 **	0.017	-		60.58	12.84
6. Sex	0.036	0.008	0.006	0.006	−0.195 ***	-		

Note: ** *p* < 0.01 *** *p* < 0.001.

**Table 3:** Bootstrap Estimates for Moderated Mediation Effects.

Conditional Indirect Effects of Distress on PTSS at Values of the Moderators (Mediated by Symptom Score)					
Social Support	Age	Effect	Boot *SE*	95% Boot CI	
				LL	UL
	47.7334	0.1820	0.0317	0.1246	0.0855
−1 *SD*	60.5773	0.1411	0.0261	0.0955	0.1178
	73.4212	0.1041	0.0273	0.0588	0.1690
	47.7334	0.2278	0.0243	0.1812	0.2757
MEAN LEVELS	60.5773	0.1718	0.0187	0.1357	0.2102
	73.4212	0.1197	0.0258	0.0723	0.1748
	47.7334	0.2354	0.0352	0.1664	0.3053
+1 *SD*	60.5773	0.1643	0.0286	0.1100	0.2197
	73.4212	0.0972	0.0355	0.0326	0.1702

Note: Relationships are statistically significant when the confidence interval does not contain zero.

## Data Availability

Data is a private data set not available publically.
